# Chest CT-based differential diagnosis of 28 patients with suspected corona virus disease 2019 (COVID-19)

**DOI:** 10.1259/bjr.20200243

**Published:** 2020-06-02

**Authors:** Sidong Xie, Ziying Lei, Xiuzhen Chen, Weimin Liu, Xiaohong Wang, Yunxu Dong, Yuefei Guo, Yani Duan, Huijuan Cao, Jie Qin, Bingliang Lin

**Affiliations:** 1 Department of Radiology, The Third Affiliated Hospital of Sun Yat-sen University, Guangzhou, China; 2 Department of Infectious Disease, The Third Affiliated Hospital of Sun Yat-sen University, Guangzhou, China

## Abstract

**Objectives:**

The chest CT findings that can distinguish patients with corona virus disease 2019 (COVID-19) from those with clinically suspected COVID-19 but subsequently found to be COVID-19 negative have not previously been described in detail. The purpose of this study was to determine the distinctions among patients with COVID-19 by comparing the imaging findings of patients with suspected confirmed COVID-19 and those of patients initially suspected to have COVID-19 who were ultimately negative for the disease.

**Methods:**

28 isolated suspected in-patients with COVID-19 were enrolled in this retrospective study from January 22, 2020 to February 6, 2020. 12 patients were confirmed to have positive severe acute respiratory syndrome corona virus 2 (SARS-CoV-2) RNA results, and 16 patients had negative results. The thin-section CT imaging findings and clinical and laboratory data of all the patients were evaluated.

**Results:**

There were no significant differences between the 12 confirmed COVID-19 (SARS-Cov-2-positive) patients and 16 SARS-CoV-2-negative patients in epidemiology and most of the clinical features or laboratory data. The CT images showed that the incidence of pure/mixed ground-glass opacities (GGOs) was not different between COVID-19 and SARS-CoV-2-negative patients [9/12 (75.0%) *vs* 10/16 (62.5%), *p* = 0.687], but pure/mixed GGOs in the peripheral were more common in patients with COVID-19 [11/12 (91.7%) *vs* 6/16 (37.5%), *p* = 0.006]. There were no significant differences in the number of lesions, bilateral lung involvement, large irregular/patchy opacities, rounded opacities, linear opacities, crazy-paving patterns, halo signs, interlobular septal thickening or air bronchograms.

**Conclusions:**

Although peripheral pure/mixed GGOs on CT may help distinguish patients with COVID-19 from clinically suspected but negative patients, CT cannot replace RT-PCR testing.

**Advances in knowledge:**

Peripheral pure/mixed GGOs on-chest CT findings can be helpful in distinguishing patients with COVID-19 from those with clinically suspected COVID-19 but subsequently found to be COVID-19 negative.

## Introduction

Corona virus disease 2019 (COVID-19), caused by SARS-CoV-2, was first reported in Wuhan, China on December 31, 2019, and rapidly spread to various places.^1–3^ By April 06, the disease had affected 206 countries, with more than 1,293,560 confirmed patients and 70,645 deaths.

Currently, positive SARS-CoV-2 RNA findings by reverse-transcriptase polymerase chain reaction (RT-PCR) is considered the gold standard for diagnosing COVID-19, but this test is time-consuming, and a shortage of supply test kits may not meet the demand for testing a large number of patients with suspected COVID-19. Furthermore, the SARS-CoV-2 RNA findings may be falsely negative due to laboratory error or insufficient specimens.^4^ Therefore, it is necessary to find a rapid and accurate method for the differential diagnosis of suspected COVID-19 patients.

Radiological examination, especially thin-section chest CT, is a non-invasive, quick method and has the advantages of discovering lung pathological changes early and reflecting the severity of the disease. Some previous radiographical studies of chest CT findings in patients with COVID-19 indicate that bilateral ground-glass opacities (GGOs) or consolidation on-chest CT should remind radiologists to make a possible diagnosis of COVID-19.^5^ However, all these data came from confirmed patients, and the characteristics to be aware of in suspected patients are not clear.

In this study, we aimed to discover the key points for diagnosis by comparing the chest CT characteristics of 12 patients with confirmed COVID-19 and 16 clinically suspected but SARS-CoV-2-negative patients.

## Methods and materials

### Patients

**Figure 1.**
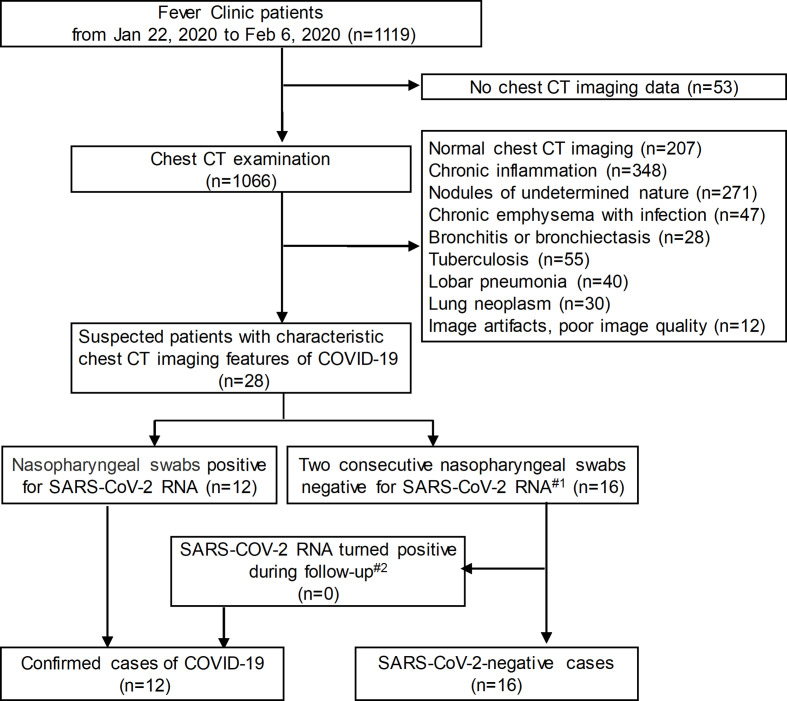
Flowchart of the study population. Note: #1 The interval between two times was more than 24 h; #2 The last follow-up was on February 20, 2020; CT = computed tomography; COVID-19 = corona virus disease 2019; SARS-CoV-2=severe acute respiratory syndrome corona virus 2.

This retrospective study was approved by the Ethic Committees of the Third Affiliated Hospital of Sun Yat-Sen University. As a retrospective study, informed consent was waived. We included all patients admitted to the Third Affiliated Hospital of Sun Yat-Sen University in Guangzhou from January 22, 2020 to February 6, 2020 (Figure 1). Our inclusion criteria (suspected patients) were patients with any epidemiological risk history and any two of the clinical characteristics as follows: (1) epidemiological history within 14 days prior to the onset of the disease: history of travel to or residence in Wuhan and its surrounding areas; close contact with COVID-19 patients or other patients from Wuhan with fever; clustered cases; and (2) clinical characteristics: fever and/or respiratory symptoms, imaging findings of pneumonia, normal or decreased total white blood cell count, or decreased lymphocyte count in the early stages of disease. Patients were confirmed by SARS-COV-2 RNA detection with respiratory or blood samples by the Guangdong Centers for Disease Control and Prevention (CDC). If the SARS-COV-2 RNA test was negative twice more than 24 h apart, the suspected patient was defined as negative. The data on demographic, epidemiological, clinical and laboratory characteristics were abstracted from medical records.^6^ A total of 1119 patients were screened in the fever clinic at the Third Affiliated Hospital of Sun Yat-Sen University, and 28 suspected patients were enrolled, including 12 confirmed and 16 negative patients.

### Chest CT

All these patients performed thin-section CT scans. The median duration from the onset of initial symptoms to CT scan was 4 days, ranging from 1 day to 2 weeks. All CT examinations were performed using an Aquilion ONE scanner (Toshiba Medical Systems; Tokyo, Japan) or an IQon Spectral scanner (Philips Healthcare, Best, the Netherlands). All CT images were obtained with the patient in the supine position during end-inspiration and without contrast medium. The CT protocol is as follows: 120 kV; automatic tube current (180 mA–440 mA); iterative reconstruction technique; detector, 32/160 mm; rotation time, 0.5 sec; section thickness, 1 mm; collimation, 0.5 mm; pitch, 1.5; and matrix, 512*512. An image reconstruction kernel was used to smooth the lung with a thickness of 1 mm and an interval of 1 mm. The CT images were viewed with lung (window width, 1,600 HU; window level, −600 HU) and mediastinal (window width, 300 HU; window level 40 HU) window settings.

All thin-section CT images were reviewed by two experienced cardiothoracic radiologists (SidongXie and Xiuzhen Chen) using a viewing console. The images were reviewed independently and reached a decision in consensus. In case of disagreements between the two primary radiologists’ interpretations, a third cardiothoracic radiologist with 20 years of experience (Jie Qin) adjudicated a final decision.

The major CT findings were described using international standard nomenclature defined by the Fleischner Society glossary and peer-reviewed literature on viral pneumonia.^7–10^ In all 28 patients, the initial CT findings were evaluated for the following features: (1) type of opacity: pure/mixed GGO or consolidation; (2) lesion location: peripheral pulmonary/subpleural or central distribution/scattered; (3) morphology of lesions: large irregular/patchy opacities, rounded opacities or linear opacities; (4) the number of lung lobes affected by GGOs or consolidation and whether there was bilateral lung involvement; (5) interstitial involvement of the lung: peribronchovascular interstitial thickening, interlobular septal thickening, tree-in-bud pattern, honeycombing, subpleural curvilinear line, and pleural thickening; (6) crazy-paving pattern; (7) halo sign; (8) air bronchogram; (9) lymphadenopathy (lymph node size of ≥10 mm in short-axis diameter); and (10) underlying lung diseases such as emphysema or bronchiectasis; and (11) other abnormalities (*e.g.,* solid pulmonary nodule, cavitation and pleural effusion).

### Statistical analysis

All data were statistically analysed using SPSS v.22.0 (IBM Corp, USA). Continuous variables are expressed as the mean (SD) or median (IQR) and were compared with the t-test or Mann-Whitney U test; categorical variables are expressed as frequency (%) and were compared by χ² test or Fisher’s exact test between the confirmed and suspected but negative patients. *P* values < 0.05 were considered to be a statistically significant difference.

## Results

### Baseline characteristics, clinical features and laboratory data

All 28 clinically suspected patients admitted to The Third Affiliated Hospital of Sun Yat-Sen University were enrolled, including 12 confirmed patients with COVID-19 and 16 SARS-CoV-2-negative patients. The confirmed patients included six males and six females; the median age was 40 years; in contrast, four men and twelve females with a median age of 35 years composed the suspected but negative patients. Patients in both groups mainly manifested fever and cough. The confirmed COVID-19 patients had more myalgia (91.6% *vs* 31.2%, *p* = 0.002), and the median number of neutrophils in SARS-CoV-2-negative patients was higher than that in confirmed patients (3.2 × 10^9^/L *vs* 4.6 × 10^9^/L,*p* < 0.05).The baseline characteristics, epidemiological factors, clinical features and laboratory data of the 12 confirmed COVID-19 patients and 16 SARS-CoV-2-negative patients are shown in Table 1.

**Table 1.** Comparison of the clinical and laboratory features between confirmed patients with COVID-19 and SARS-CoV-2-negative patients

**Characteristics**	Confirmed patients (*N* = 12)	Negative patients (*N* = 16)	*P* values
Age, years, median (IQR)	40 (30–61）	35 (21–53)	0.265
Sex			0.243
Females(n, %)	6 (50.0)	4 (25.0)
Males(n, %)	6 (50.0)	12 (75.0)
Chronic medical illness(n, %)	4 (33.3)	4 (25.0)	0.691
**Signs and symptoms**			
Fever(n, %)	12 (100)	13 (81.2)	0.238
Cough(n, %)	9 (75.0)	15 (93.7)	0.285
Shortness of breath(n, %)	2 (16.6)	2 (12.5)	1
Myalgia(n, %)	11 (91.6)	5 (31.2)	**0.002**
Sore throat(n, %)	1 (8.3)	2 (12.5)	1
Diarrhoea(n, %)	2 (16.6)	4 (25.0)	0.673
Nausea and vomiting(n, %)	3 (25.0)	3 (18.75)	1
**Epidemiological survey**			
History of stay in Wuhan(n, %)	8 (66.6)	4 (25.0)	0.053
Contact with Wuhan residents(n, %)	11 (91.6)	10 (62.5)	0.184
Cluster outbreak(n, %)	6 (50.0)	4 (25.0)	0.243
**Blood routine**			
Leucocytes (×10^9^/L, normal range 3.5–9.5)	4.9 (4.3–6.9)	6.8 (5.4–9.1)	0.095
Neutrophils (×10^9^/L, normal range 1.8–6.3)	3.2 (2.7–3.8)	4.6 (3.4–6.1)	**0.029**
Lymphocytes (×10^9^/L, normal range 1.1–3.2)	1.2 (1.0–1.7)	1.0 (0.7–1.8)	0.246
Platelets (×10^9^/L, normal range 125.0–350.0)	189.5 (144.7–223.7)	197.5 (154.7–253.2)	0.546
Haemoglobin (g/L, normal range 130.0–175.0)	143.0 (133.5–149.7)	138.5 (123.2–148)	0.416
**Blood biochemistry**			
Alanine aminotransferase (U/L, normal range 3.0–35.0)	14.5 (12.2–23.7)	23.0 (14.5–50.7)	0.21
Total bilirubin (μmol/L, normal range 4.0–23.9)	7.3 (4.9–11.6)	8.1 (6.1–15.2)	0.39
Albumin (g/L, normal range 36.0–51.0)	47.5 (41.5–49.0)	44.5 (34.6–49.8)	0.21
Erythrocyte sedimentation rate (mm/h, normal range 0–15)	12.5 (7.0–30.5)	13.0 (6.0–35.0)	0.807
C-reaction protein (mg/L, normal range 0–6)	8.1 (6.7–28.2)	13.5 (2.2–106.8)	0.889
Procalcitonin (ng/mL, normal range 0–0.05)	0.03 (0.02–0.06)	0.08 (0.03–0.19)	0.064

Data are n (%) and median(IQR). IQR=interquartile range. *N* is the total number of patients withavailable data. SARS-CoV-2=severe acuterespiratory syndrome coronavirus 2. *P* values for the comparisons between two groups were derived using Fisher’s exact test for categorized variables and the Mann-Whitney U test for continuous variables.

### CT imaging findings

The initial thin-section chest CT findings of 28 clinically suspected patients with COVID-19 are presented in Table 2. The main chest CT findings of clinically suspected patients included bilateral multi-focal lesions in the lungs, halo signs and pure/mixed GGOs, which were more frequently located in the peripheral pulmonary/subpleural areas in the confirmed COVID-19 patients than in the suspected but negative patients (91.7% *vs* 37.5%, *p* = 0.006).

**Table 2.** Comparison of the frequencies of CT features between confirmed patients with COVID-19 and SARS-CoV-2-negative patients

Feature	Confirmed patients (*N* = 12)	Negative patients (*N* = 16)	*P* values
Type of exudative lesions			0.687
Pure/mixed GGO	9 (75.0)	10 (62.5)	
Consolidation	3 (25.0)	6 (37.5)	
Lesion location			**0.006**
Peripheral/subpleural	11 (91.7)	6 (37.5)	
Central distribution/scattered	1 (8.3)	10 (62.5)	
Number of lesions			0.429
Multifocal	11 (91.7)	16 (100.0)	
Unifocal	1 (8.3)	0 (0.0)	
Bilateral lung involvement	10 (83.3)	10 (62.5)	0.401
Morphology of lesions			
Large irregular/patchy opacities	12 (100.0)	16 (100.0)	1.000
Rounded opacities	4 (33.3)	10 (62.5)	0.127
Linear opacities	7 (58.3)	7 (43.8)	0.445
Crazy-paving pattern	5 (41.7)	4 (25.0)	0.432
Halo sign	9 (75.0)	8 (50.0)	0.253
Air bronchogram	5 (41.7)	7 (43.8)	0.912
Peribronchovascular interstitial thickening	6 (50.0)	6 (37.5)	0.508
Interlobular septal thickening	8 (66.7)	6 (37.5)	0.127
Tree-in-bud pattern	4 (33.3)	6 (37.5)	1.000
Subpleural curvilinear line	2 (16.7)	0 (0.0)	0.175
Honeycombing	0 (0.0)	0 (0.0)	1.000
Pleural thickening	2 (16.7)	1 (6.2)	0.560
Solid pulmonary nodule	4 (33.3)	1 (6.2)	0.133
Pleural effusion	0 (0.0)	3 (18.8)	0.238
Cavitation	0 (0.0)	0 (0.0)	1.000
Lymphadenopathy	0 (0.0)	1 (6.2)	1.000
Emphysema	0 (0.0)	0 (0.0)	1.000
Bronchiectasis	2 (16.7)	3 (18.8)	1.000

Data are *n* (%), *N* is the total number of patients with available data. Percentages may not add up to 100% because of rounding. *P* values for the comparisons between two groups were derived using Fisher’s exacttest for categorized variables. GGOs = ground-glass opacities; COVID-19 =coronavirus disease 2019; SARS-CoV-2 = severe acute respiratory syndrome coronavirus 2.

**Figure 2.**
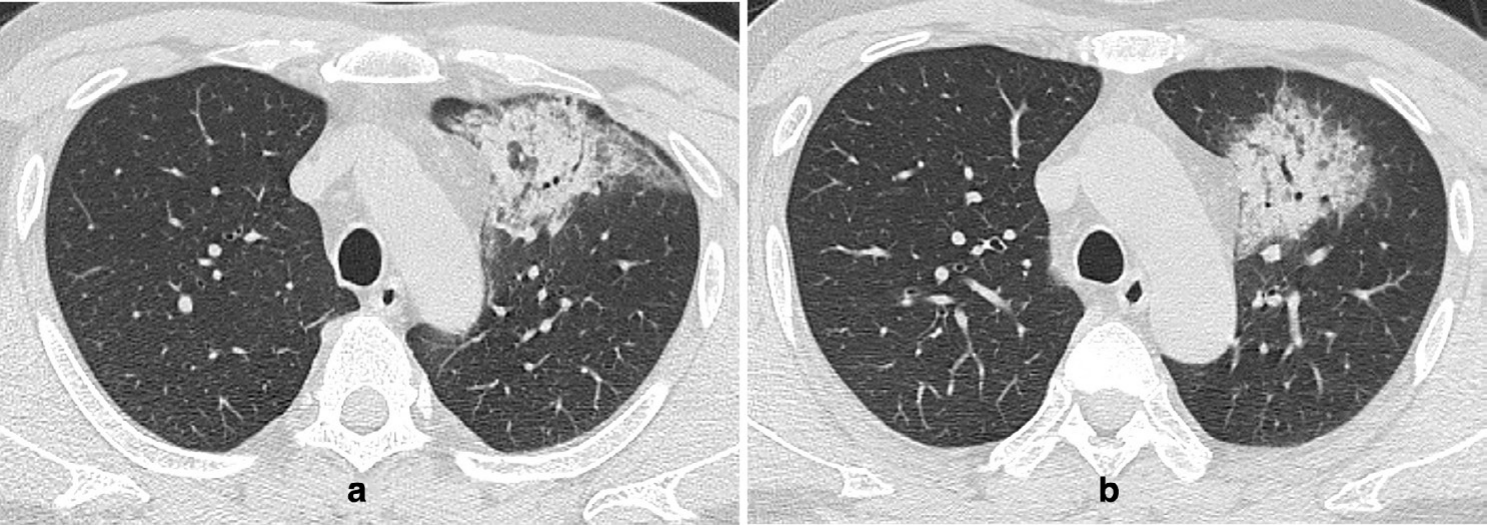
(a-b) An axial CT image obtained without intravenous contrast in a 43-year-old male showed peripheral/subpleural patchy consolidation in the anterior of the left upper lobe, with interlobular septal thickening, air bronchogram and linear opacities. He had a history of living in Xiaogan, Hubei Province and presented with fever for 1 week. Nasopharyngeal swabs for SARS-CoV-2 RNA were positive.

**Figure 3.**
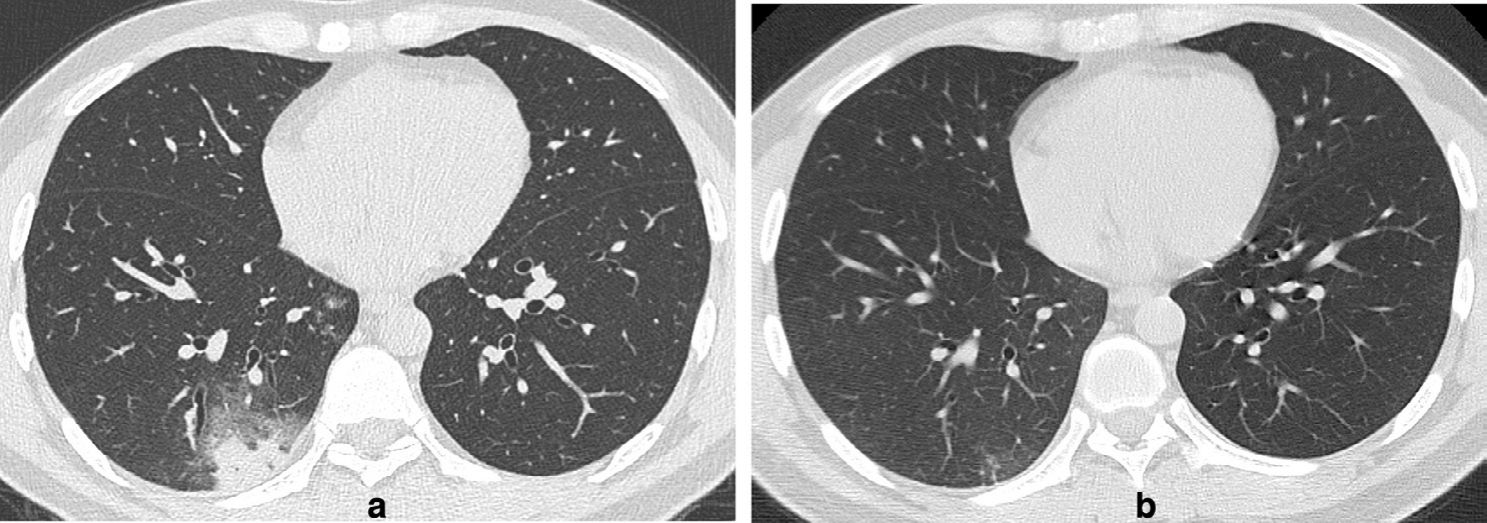
(a-b) A31-year-old male with a history of travelling on a cruise presented with fever, diarrhoea, sore throat and fatigue. (a) Axial thin-section non-contrast CT image on February 1, 2020, showed peripheral/subpleural, patchy, rounded, mixed GGOs in the right lower lobe, with interlobular septal thickening and crazy-paving patterns. (b) Follow-up CT image on February 9, 2020, showed that the lesions were obviously resolved after anti-infection therapy. Nasopharyngeal swabs for SARS-CoV-2 RNA were negative.

**Figure 4.**
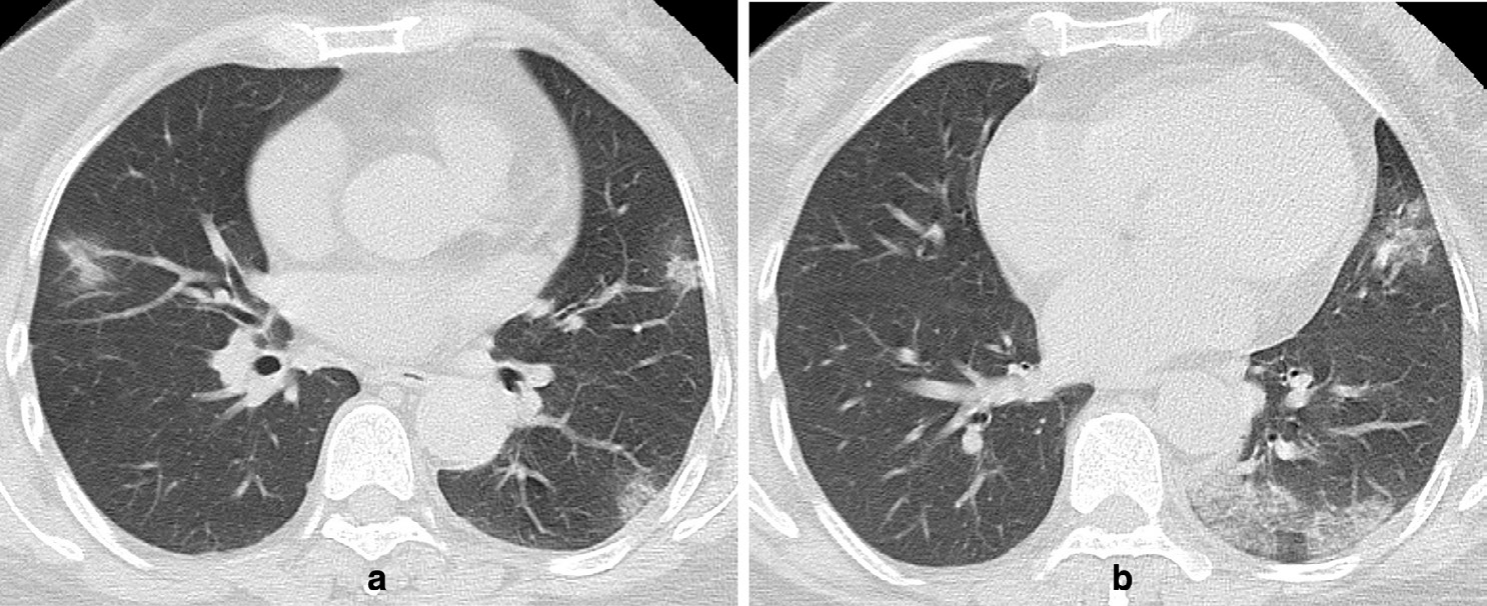
(a-b) A 56-year-old female with a history of travelling to Guangzhou from Wuhan presented with fever, cough and diarrhoea for 2 days. (a) (b) Axial thin-section non-contrast CT image showed bilateral peripheral/subpleural, patchy, pure/mixed GGOs with interlobular septal thickening and crazy-paving patterns. Two consecutive nasopharyngeal swabs for SARS-CoV-2 RNA(the interval between the two swabs more than 24 h) were negative.

The number of lesions, bilateral lung involvement, large irregular/patchy opacities, rounded opacities and linear opacities, crazy-paving patterns, interlobular septal thickening, air bronchograms, peribronchovascular interstitial thickening, tree-in-bud patterns, subpleural curvilinear lines and pleural thickening were apparent in confirmed and suspected but negative patients, and there were no significant differences. (Figures 2–4)

## Discussion

To screen for patients with COVID-19 and control the spread of the epidemic, clinically suspected patients need to be isolated. Chest imaging findings are important criteria for suspected cases. In our study, clinically suspected patients had bilateral multi-focal lung lesions, halo signs and pure/mixed GGO opacities, which were more peripheral/subpleural in the confirmed COVID-19 patients. Because COVID-19 mainly involves the respiratory system, chest CT is more sensitive than chest X-ray (CXR) in differential diagnosis initial evaluation, response evaluation and follow-up of COVID-19. CXR is usually no abnormal findings in the early stage of COVID-19, whereas CT findings may be present even before symptom onset.^11,12^ Furthermore, CT imaging have been proven to have diagnostic value in several studies with initially false-negative RT-PCR screening results.^4,13,14^ Therefore, to identify COVID-19 patients early and control the source of the infection, CT is often a first-line investigation for COVID-19 in mainland China. Many studies have concluded that the CT findings of COVID-19 include bilateral pulmonary parenchymal pure/mixed GGOs in the lung periphery, accompanying crazy-paving patterns, consolidation, intralobular interstitial thickening and interlobular septal thickening^3-5, 15, 16^. The common CXR findings are similar to those described for CT: bilateral, peripheral, consolidation and/or GGOs.^12,15^ when the disease developed from the intermediate to rapid progression stages, CXR may show bilateral lung diffuse consolidative opacities—the features of acute respiratory distress syndrome (ARDS).^12^ However, unlike in confirmed COVID-19 patients, whether these imaging findings exist in the suspected but negative patients has not been reported. It is clinically important to identify negative patients from suspected COVID-19 patients.

In our study, pure/mixed GGOs were also common in negative patients. Many pulmonary infectious diseases, such as influenza virus pneumonia, and non-infectious conditions, including interstitial pulmonary oedema, pulmonary haemorrhage, respiratory bronchiolitis, hypersensitivity pneumonitis, organising pneumonia and alveolar proteinosis, can show similar imaging manifestations.^8,9^ In addition, the type of exudative lesions is related to the different stages of COVID-19 pneumonia. Pan et al^16^ reported that typical mild COVID-19 pneumonia initially presented as small subpleural, unilateral or bilateral GGOs in the lower lobes, which then changed into crazy-paving patterns and subsequent consolidation in a few days.

However, pure/mixed GGOs in the peripheries was a special imaging feature for differential diagnosis to distinguish patients with COVID-19 from clinically suspected patients in our study. The lesions of COVID-19 patients were predominately distributed in the peripheral/subpleural pulmonary regions.^3,16,17^ We speculate that this is related to the pathophysiological mechanism of the disease. COVID-19 is caused by SARS-CoV-2, which is approximately 50–200 nm in diameter and is prone to staying in the terminal bronchioles and causing lung damage. In addition, SARS-CoV-2 mainly invades cells containing angiotensin-converting enzyme 2 (ACE2) receptors, which are found in regions rich in bronchial epithelial cells and type 2 alveolar epithelial cells, especially the latter.^18–20^As a result, the peripheral lung is attacked first and suffers the most damage. Nevertheless, the GGOs of both H1N1 pneumonia and SARS are also distributed more peripherally.^21,22^ Therefore, the single factor of peripheral/subpleural lung distribution in COVID-19 is not very unique.

In addition, other CT features, including interlobular septal thickening, crazy-paving patterns, halo signs and consolidation, were not specific for a diagnosis of COVID-19 and could be seen in the negative patients. The crazy-paving pattern has been shown to occur in many other diseases, such as usual interstitial pneumonia, infection, pulmonary oedema, haemorrhage, ARDS, alveolar proteinosis, bronchiolitis obliterans organising pneumonia (BOOP), andradiation pneumonitis.^10,23^

In our study, more confirmed COVID-19 patients had myalgia, but there were no differences between the two groups patients in other clinical symptoms. The neutrophil count in SARS-CoV-2-negative patients was higher than that in confirmed patients. This suggests the possibility of bacterial infection in suspected but negative patients, which may be an identifying point.

Because the clinical features and imaging findings are not unique to confirmed patients with COVID-19, aetiologic evidence, including SARS-CoV-2 RNA and serum IgM antibodies, remains the diagnostic standard for COVID-19.

There are several limitations to our study. First, the sample size of confirmed and suspected but negative patients was small. Second, the pathogens of most patients who were SARS-CoV-2 negative were not clear. Third, there were no pathological results of lung tissue available to investigate the correlation between radiological and histopathological findings.

In conclusion, although peripheral pure/mixed GGOs on CT may help distinguish patients with COVID-19 from clinically suspected but negative patients, CT cannot replace RT-PCR testing.
